# Workers *v.*, Beggars

**Published:** 1887-02-05

**Authors:** 


### WORKERS *V.* BEGGARS.

"He who drives fat oxen should himself be fat;" and they who administer the funds of charity should themselves be possessed with the charitable spirit. We are anxious on the present occasion to turn the thoughts of Hospital Managers in a backward direction, and to ask them to give their most serious consideration to those who supply the resources for hospital work. The subscribers and donors to public institutions are more or less at the mercy of committees and employés. They know that sickness, sorrow and poverty are all around them; and a noble spirit of sympathy and brotherhood prompts them to make efforts, sometimes involving great self-sacrifice, to relieve the sufferings of the poor and sick. Such a spirit on the part of donors and subscribers should evoke a similar spirit in all hospital officials, from the chairman of committee to the humblest probationer on the nursing staff.

Hospital Managers have the administration of immense sums of money. How are these obtained? In the majority of cases by an organised system of begging; and the hospital which is the least scrupulous and the most energetic in its begging appeals generally finds itself possessed of the most abundant resources. We do not say that no begging should be done; but we do say that charities, even voluntary charities, should beg as little as possible; and we venture to offer some suggestions which we think will tend to diminish begging in a very considerable degree. We are about to make what some may regard as a very unjustifiable assertion, but we will make it nevertheless. It is this: If the English people could be convinced that every shilling given in charity produced its full equivalent of truly charitable work; or, in other words, that none of the money was wasted in extravagance or by unskilful management, there would be no necessity for begging at all. So great is the sympathy of the public with sickness when it is associated with hopeless want, that they would, unsolicited, supply sufficient resources for all the really necessitous cases of sickness and accident. We think we are warranted in making this assertion by the history of the Bristol Orphanage and other institutions which might be named. Once let the public be imbued with full confidence in the economic and skilful management of a charitable organisation and that organisation will never want funds.

The Managers of the Newcastle Infirmary, as we pointed out in an article last week, and as is shewn by the Supplement published to-day, have set their hands resolutely to the work of investigation and retrenchment. Their enquiries into the expenditure of the hospital have been of the most minute and far-reaching character. As a result they have been able to place before their subscribers and the public a report which presents, as in a picture, every detail of cost and administration for a definite period. The report proves beyond a doubt, that though no great amount of fault can be charged upon the management as a whole; yet if those who have had the spending of the money in every department had spent it as they would have expended their own private means, the Newcastle Infirmary to-day would not have required to appoint a sub-committee to inquire into the question of expenditure. That is the great point we desire to emphasise here and now. One or two plain questions may be permitted. How many of those who have administered the metropolitan and country hospitals have spent the money of the hospitals as they would have spent their own? If they had so spent it, would the hospitals have now been in such serious financial straits? These are home questions. But the subject must be dealt with in the most thorough and searching manner. There is a limit to the efficacy of begging, but no limit to the growing numbers of the sick. If the hospitals are to keep pace with their proper work some other means than ceaseless appeals to a wearied and nauseated public must be found.

Instead of eternally *begging*, we suggest a change to *working*. What is the *cause* of the chronic poverty of hospitals? Let that be made the subject of radical enquiry. This is the first thing to be done. Let each and all the hospital committees and officials ask and ascertain, is there mismanagement or waste anywhere, and where, in our hospital? Is it in the kitchen or in the wards, in the board-room or in the secretary's office? Is it in food, or in physic, in instruments or in stimulants? Wherever it is, and however large or small the amount, let it be brought to light, or, if there be neither waste nor mismanagement, then let it be proved by the publication of the facts on the lines of the Newcastle report. Nothing can be done without thoroughness. Do members of hospital committees say they have not time or patience to devote to such enquiries? Then why do they belong to such committees? Let them stand aside and give place to others, who will devote such time and labour to the conscientious discharge of their duties as the gravity of the occasion demands.

The contrast between the annual cost per bed in different hospitals after every allowance has been made for varying circumstances and conditions of management is startling. It is not too much to say that some hospitals spend almost twice as much upon each patient annually as others, without producing any better results, or showing any justification whatever for their extravagant expenditure of charitable resources. This certainly cannot continue for ever. Just as an overtaxed nation reaches a point when it refuses to be taxed any further or at all, so will the burdened philanthropic public sooner or later throw off its self-imposed load, and leave the extravagantly-conducted hospitals to sink or swim as best they can.

A Royal Commission is suggested, and small wonder. But what Royal Commission could do half the service for hospitals that the several committees could do for them individually if they would? A Royal Commission is equivalent to a general confession of incapacity all round on the part of Hospital Managers. If such a Commission were appointed it would do nothing but collect evidence and offer suggestions. What a wretched futility that would be! We have now nearly completed an exhaustive analysis of the expenditure of all the British hospitals, but before publishing it we desire to give the managers of each institution an opportunity of looking into this question of expenditure, for themselves, and with this object we offer the prizes mentioned on p. 318. It is not suggestions only that are wanted, but workers; men who will regard the affairs of their several hospitals as they would consider their own. Half-a-dozen such men on each hospital committee would produce a full exchequer in five years.

